# Resting-State Functional Connectivity Associated With Extraversion and Agreeableness in Adolescence

**DOI:** 10.3389/fnbeh.2021.644790

**Published:** 2022-01-03

**Authors:** Leehyun Yoon, Angelica F. Carranza, Johnna R. Swartz

**Affiliations:** Department of Human Ecology, University of California, Davis, Davis, CA, United States

**Keywords:** adolescence, extraversion, agreeableness, amygdala, functional connectivity

## Abstract

Although adolescence is a period in which developmental changes occur in brain connectivity, personality formation, and peer interaction, few studies have examined the neural correlates of personality dimensions related to social behavior within adolescent samples. The current study aims to investigate whether adolescents’ brain functional connectivity is associated with extraversion and agreeableness, personality dimensions linked to peer acceptance, social network size, and friendship quality. Considering sex-variant neural maturation in adolescence, we also examined sex-specific associations between personality and functional connectivity. Using resting-state functional magnetic resonance imaging (fMRI) data from a community sample of 70 adolescents aged 12–15, we examined associations between self-reported extraversion and agreeableness and seed-to-whole brain connectivity with the amygdala as a seed region of interest. Then, using 415 brain regions that correspond to 8 major brain networks and subcortex, we explored neural connectivity within brain networks and across the whole-brain. We conducted group-level multiple regression analyses with the regressors of extraversion, agreeableness, and their interactions with sex. Results demonstrated that amygdala connectivity with the postcentral gyrus, middle temporal gyrus, and the temporal pole is positively associated with extraversion in girls and negatively associated with extraversion in boys. Agreeableness was positively associated with amygdala connectivity with the middle occipital cortex and superior parietal cortex, in the same direction for boys and girls. Results of the whole-brain connectivity analysis revealed that the connectivity of the postcentral gyrus, located in the dorsal attention network, with regions in default mode network (DMN), salience/ventral attention network, and control network (CON) was associated with extraversion, with most connections showing positive associations in girls and negative associations in boys. For agreeableness, results of the within-network connectivity analysis showed that connections within the limbic network were positively associated with agreeableness in boys while negatively associated with or not associated with agreeableness in girls. Results suggest that intrinsic functional connectivity may contribute to adolescents’ individual differences in extraversion and agreeableness and highlights sex-specific neural connectivity patterns associated with the two personality dimensions. This study deepens our understanding of the neurobiological correlates of adolescent personality that may lead to different developmental trajectories of social experience.

## Introduction

Understanding the neural correlates of individual differences in extraversion and agreeableness in adolescence is important because these two personality dimensions seem to have a critical role in shaping favorable peer relationships. Extraversion and agreeableness are characterized by high motivation to create and engage in social interaction (Elphick et al., 1998) and high motivation to maintain positive social relationships and achieve interpersonal intimacy (Costa and McCrae, 1992; Jensen-Campbell and Graziano, 2001), respectively. Both personality traits have been associated with a high level of peer acceptance (Jensen-Campbell et al., 2002; Lubbers et al., 2006; Van der Linden et al., 2010; Wolters et al., 2014; Szczygiel and Mikolajczak, 2018), high level of friendship quality (Jensen-Campbell et al., 2002), and low level of loneliness (Vanhalst et al., 2013). Moreover, higher extraversion and agreeableness are associated with a larger social network. Extraverted adolescents select many peers as friends, and agreeable adolescents are often selected as friends by many peers (Selfhout et al., 2010). Additionally, agreeableness is positively associated with peer nominations of liking (Lopes et al., 2005) and protects vulnerable adolescents from experiencing peer victimization (Jensen-Campbell et al., 2002). Beyond their role in forming positive peer relationships, extraversion and agreeableness during adolescence have also been associated with reduced psychosocial problems such as internalizing (Jensen-Campbell et al., 2002; Klimstra et al., 2010), externalizing problems (Jensen-Campbell et al., 2002), general psychopathology factor (Castellanos-Ryan et al., 2016), self-injury (You et al., 2016), and problematic social media use (Marino et al., 2016), further indicating the significance of elucidating the neural correlates of variability in these two personality traits in adolescence.

Research has found several temperamental, environmental, and neural correlates of extraversion and agreeableness in adolescence. Higher extraversion in adolescence was found to be associated with lower shyness in infancy and lower growth in shyness (Baardstu et al., 2020), warm parenting (De Haan et al., 2013), and lower cortisol reactivity (Evans et al., 2016). In girls, high extraversion, specifically, the positive emotionality facet, was associated with increased late positive potential (LPP) to pleasant and unpleasant stimuli, which reflects sustained attention (Speed et al., 2015). Some results suggest more nuanced patterns of findings. For example, high levels of the positive emotionality facet of extraversion in adolescent girls combined with lower levels of neuroticism were associated with increased reward positivity, an event-related potential (ERP) component that reflects sensitivity to monetary reward (Speed et al., 2018). In addition, extraversion was associated with gray matter volume of medial frontal gyrus in the opposite direction for boys and girls (Blankstein et al., 2009), with a positive association between extraversion and volume in boys and a negative association in girls. Higher benevolence in adolescence, a measurement corresponding to agreeableness, was observed to be associated with lower overreactive parenting (De Haan et al., 2013) and lower surface area in the right occipital lobe (Ferschmann et al., 2018). As such, the literature has begun to document the potential antecedents or correlates of adolescent extraversion and agreeableness. However, surprisingly, no study has related adolescent extraversion and agreeableness to resting-state functional connectivity (rsFC), a neural measurement that has received remarkable attention in personality neuroscience due to its potential utility to predict personality (Markett et al., 2018).

Studies with adult samples have accumulated evidence of the association between rsFC with extraversion and agreeableness. Patterns of rsFC associated with extraversion have been heterogeneous across studies: Extraversion was found to be associated with connectivity within the default mode network (DMN) (Sampaio et al., 2014), connectivity within the salience network (Tian et al., 2018), and amygdala connectivity with regions responsible for emotional and visual processing such as the temporal pole, insula, putamen, and occipital cortex (Aghajani et al., 2014; Pang et al., 2016). Another study found that the motor cortex and visual cortex’s connectivity with other brain regions predict extraversion (Hsu et al., 2018). Meanwhile, a study (Nostro et al., 2018) demonstrated that face perception network and reward network connectivity predicted extraversion in males and females, respectively. Consistent with findings of sex differences in the neural correlates of extraversion, one study using task fMRI in an adult sample found that the warmth facet of extraversion was negatively associated with amygdala activity in women, but not significantly associated with amygdala activity in men (Swartz et al., 2017b).

Intrinsic functional connectivity associated with agreeableness has been relatively consistent across studies: In line with its role in interpersonal functioning, agreeableness has been associated with connectivity involving the DMN, the brain network implicated in social memory, perspective-taking, and self-referential processing (Adelstein et al., 2011; Ryan et al., 2011; Sampaio et al., 2014; Xiao et al., 2019). Moreover, a recent study found that the DMN was a primary contributor in predicting agreeableness from the whole-brain functional connectome (Cai et al., 2020). In addition, a study (Nostro et al., 2018) found that connectivity between the DMN and Mirror neuron network (implicated in simulating others’ actions and emotions) predicts agreeableness. Relatedly, a study with a wide age range of adult samples (Simon et al., 2020) found that agreeableness is associated with reduced within-network connectivity of the dorsal attention (DA) network, the task-positive brain network known to have anticorrelation with the DMN during rest (Fox et al., 2005).

Despite such a considerable amount of studies linking rsFC with extraversion and agreeableness in adult samples, separate investigation within adolescent samples is necessary. Due to the dynamic functional brain reorganization that occurs during adolescence (Stevens, 2016; van Duijvenvoorde et al., 2019), unique patterns of rsFC underlying personality in adolescents may be observed. The current study aimed to explore the association between rsFC, extraversion, and agreeableness in 70 adolescents aged 12–15. We also examined whether rsFC correlated with the two personality traits differently for girls and boys (i.e., an interaction effect of sex and personality) per recent recommendation for sex-specific analysis when examining the neural basis of personality (Nostro et al., 2018) and the observed sex differences in intrinsic functional connectivity and its development during adolescence (Alarcón et al., 2015; Satterthwaite et al., 2015; Ernst et al., 2019).

We first examined amygdala connectivity, because the amygdala and the regions anatomically and functionally connected to the amygdala (e.g., visual cortex, medial prefrontal cortex, temporal pole) have a crucial role in the integration of social cues, social approach, and prosocial behavior (Bickart et al., 2014 for a review). Moreover, high intrinsic amygdala connectivity with brain regions related to social perception (e.g., temporal pole, superior temporal sulcus) and social affiliation (e.g., medial prefrontal cortex) was associated with a larger social network (Bickart et al., 2012), which extraverted and agreeable adolescents tend to have (Selfhout et al., 2010). Beyond the amygdala connectivity analysis, to explore brain connectivity unbiased by selecting particular seed regions, we examined connectivity between all pairs of whole brain regions that correspond to 17 brain networks (Schaefer et al., 2018) and subcortex. Finally, whole-brain connectivity analysis was followed by within-network connectivity analysis that restricts the regions of interest to a single network among 8 major brain networks (e.g., DMN, dorsal attention network) subcortex. We used both whole-brain connectivity and within-network connectivity approaches to explore whether extraversion and agreeableness are associated with connectivity between any regions across the brain and/or within a specific brain network. In addition, based on an emerging literature that has suggested that changes in hormone levels throughout the female menstrual cycle are associated with rsFC (Beltz and Moser, 2020; Pritschet et al., 2020), we examined whether the female-specific findings were replicated when controlling for days since the last period as a covariate. We also examined whether the observed effect of extraversion and agreeableness on rsFC remained significant after controlling for depression and anxiety symptoms, which are often associated with extraversion and agreeableness (Klimstra et al., 2010; Lyon et al., 2021).

## Materials and Methods

### Participants

Participants aged 12–15 were recruited from the community for the Adolescent Health and Brain Study. The recruitment method and inclusion criteria for scanning were reported in detail in a previous study with the same sample (Swartz et al., 2019). Briefly, inclusion criteria were that the adolescent spoke English and was capable of understanding all study procedures and lying still in the MRI scanner; exclusion criteria included any contraindications to MRI scanning, chronic diseases or conditions that could affect cerebral blood flow, use of psychotropic medications, and presence of psychiatric disorders based on parent-report (autism spectrum disorder, attention-deficit/hyperactivity disorder, schizophrenia, or bipolar disorder). Among 105 participants who underwent fMRI scanning, 33 participants were excluded due to ending the scan early (*N* = 4), excessive head movement (*N* = 21), poor MRI data quality (*N* = 3), MRI technical problem (*N* = 1), incidental finding (*N* = 1), running out of time during the scanning session (*N* = 2), and no questionnaire data (*N* = 1). In addition, we excluded 2 participants who identified as non-binary to implement personality by sex interaction analyses. The final sample included in the analysis was 70 (Mean age = 13.64, Female *N* = 34). The study procedure was approved by the University of California, Davis IRB. Parents and adolescents provided informed consent and informed assent, respectively.

### Data Acquisition

MRI data were acquired with a 3T Siemens TIM trio MRI system located at the UC Davis Imaging Research Center. After acquiring localizer scans, a T1-weighted magnetization-prepared rapid gradient-echo (MPRAGE) sequence was applied to obtain high-resolution structural images. The following parameters were used for structural image acquisition: TR = 2,500 ms, TE = 4.33 ms, flip angle = 7, FOV = 243 mm, number of slices = 208, slice thickness = 0.95 mm, voxel size = 0.9 mm isotropic. To ensure global field homogeneity, a semi-automated high-order shimming program was used. After participants completed three tasks (not reported here), blood-oxygen-level-dependent (BOLD) images were collected during the resting state scan. The fMRI data was acquired with a gradient-echo based, echo planar imaging (EPI) sequence with the following parameters: TR = 2,000 ms, TE = 27 ms, flip angle = 80, number of slices = 35, slice order = interleaved, FOV = 224 mm, slice thickness = 3.5 mm, voxel size = 3.5 mm isotropic. For static magnetic field inhomogeneity correction, we collected a field map with slices prescribed along with the EPI slices with TE 1 = 2.46 ms, TE 2 = 4.92 ms. During the rsFC data acquisition, participants were instructed to view a white fixation cross on a black screen. We acquired 200 volumes of rsFC data for the first 13 participants reported on in this paper; for the remaining 57 participants reported on in this paper, the acquisition was shortened to 168 volumes of rsFC data to shorten the length of the scan. In the statistical analyses, we controlled for the acquired volume number as a covariate using a categorical variable (i.e., 200 volumes = 1, 168 volumes = −1).

### Preprocessing and Denoising

The data analysis was conducted using the Conn toolbox v.17.f.^1^ We applied the default Conn toolbox pre-processing pipeline, which included realignment and unwarping using the field map, slice-timing correction, detection of outlier scans, segmentation, normalization, and smoothing with a 6 mm full-width-at-half-maximum Gaussian Kernel. After preprocessing, the data were visually inspected for quality assurance, and 3 participants with poor data quality were excluded for further analysis. In addition, we ran a denoising pipeline that included 3 procedures: (1) scrubbing volumes with the conservative criteria (i.e., a global-signal *z*-value threshold of 3 or volume-to-volume motion threshold of 0.5 mm), (2) adding nuisance regressors of noise components from white matter, cerebrospinal areas, and motion parameters, specifically 3 translation, 3 rotation, and their first-order derivatives, (3) bandpass filtering of BOLD signals to 0.01–0.1 Hz window. Participants with over 15% of censored volumes were excluded for further analyses.

### Measurement for Extraversion and Agreeableness

At a separate visit before fMRI scanning, participants completed the 44-item Big Five Inventory (John et al., 1991, 2008; John and Srivastava, 1999) including 8 items for measuring extraversion and 9 items for measuring agreeableness. They were asked to rate how much they agree with each item (1: strongly disagree, 5: strongly agree). Each participant’s ratings on items were averaged to create scores for extraversion and agreeableness. The Cronbach’s alphas for extraversion and agreeableness were 0.80 and 0.73, respectively. To confirm the normality of extraversion and agreeableness scores, we conducted Shapiro Wilk test. We also examined the normality of the measurements for boys and girls separately. We explored any sex difference in extraversion and agreeableness scores.

### Amygdala Seed-Based Connectivity Analysis

Seed regions of the left and right amygdala were defined using the automated anatomical labeling 2 atlas (AAL2) (Tzourio-Mazoyer et al., 2002). The connectivity maps of left and right amygdala connectivity were estimated for each individual. To identify the regions whose connectivity with left or right amygdala are associated with extraversion, agreeableness, extraversion by sex, or agreeableness by sex, the connectivity maps were submitted to group-level multiple regression analyses with the predictors of extraversion, agreeableness, extraversion by sex, agreeableness by sex, sex (girls: -1, boys: 1), age, and scan length category (200 volumes: 1, 168 volumes: -1). For the statistical threshold, we combined the voxel-level threshold of uncorrected *p* < 0.001 with the cluster-size threshold of *p*(FDR) < 0.025 (i.e., 0.05/2 amygdala seeds).

After the regions associated with interaction terms (i.e., extraversion by sex or agreeableness by sex) were identified, and after extracting the connectivity value from CONN, we performed simple slope analyses that examined the association between extraversion or agreeableness and connectivity for boys and girls using *emtrends* function of emmeans package of R.

### ROI-to-ROI Analysis With Large-Scale Network Atlas

To explore rsFC across regions in the whole brain, we used 400 cortical regions which corresponded to 18 brain networks (Schaefer et al., 2018) which were further grouped into 8 major networks [i.e., DMN, control network (CON), somatomotor network, visual network, limbic network, salience/ventral attention network (SAL/VA), dorsal attention network (DA), and temporoparietal network]. For example, a major network of DMN includes three networks of DMN-A, DMN-B, and DMN-C. We additionally included 15 regions in the subcortex (i.e., left and right accumbens, caudate, pallidum, putamen, thalamus, amygdala, hippocampus, and brain stem) provided in the CONN toolbox. We did not include the cerebellum because the brain coverage of 6 participants did not cover this region. Please see Supplementary Figure 1 for the description of each network.

The rsFC between the BOLD time series between all pairs of 415 ROIs was estimated for each participant. We conducted multiple regression analyses with the same predictors as the amygdala connectivity analysis to identify any pairs of regions associated with extraversion, agreeableness, extraversion by sex, or agreeableness by sex. For the statistical threshold, we used connection-level threshold (*p*-uncorrected < 0.001) combined with network-based statistics (NBS) *p*(FDR) (by intensity)<0.05. Any connection with the interaction effect was further probed with simple slope analyses.

In addition to examining connections within whole-brain regions, we also conducted analyses examining connections within the 8 major brain networks and subcortex. For this analysis, rsFC between the pairs of regions within each brain network (e.g., 79 × 79 for the DMN) was estimated. The same procedures for statistical analysis and thresholding were conducted as the whole-brain connectivity analysis, except that we applied NBS *p*(FDR) (by intensity) < 0.0056 (i.e., 0.05/9) to correct for multiple tests for 8 brain networks and subcortex.

### Supplementary Analysis: Controlling for Menstrual Cycle, Depression, and Anxiety

To examine whether the female-specific associations between personality and rsFC found in the main analysis were replicated when controlling for the menstrual cycle, we conducted a *post hoc* supplementary analysis including number of days since the last period (self-reported by participants) as a covariate. We conducted linear regression analyses with the dependent variable of any patterns of rsFC found to have a significant personality by sex interaction, independent variables of extraversion and agreeableness, and covariates of days since last period, age, and scan length.

Given that extraversion and agreeableness are often negatively associated with depression and anxiety (Klimstra et al., 2010; Lyon et al., 2021), we examined the correlation between extraversion and agreeableness with depression and anxiety symptoms in our sample. When significant associations were found, we conducted a *post hoc* analysis to confirm whether the observed effect of extraversion and agreeableness on rsFC was maintained after controlling for depression or anxiety. The Center for Epidemiological Studies Depression Scale for Children (CES-DC) (Weissman et al., 1980; Faulstich et al., 1986) (scale: 0–3) and Screen for Child Anxiety Related Disorders (SCARED) (Birmaher et al., 1997, 1999) (scale: 0–2), both self-reported by the adolescent, were used to measure depression and anxiety symptoms, respectively. We calculated the mean of items for each measure.

## Results

### Descriptive Statistics of Extraversion and Agreeableness

**FIGURE 1:**
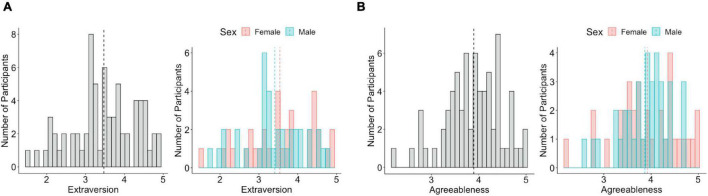
Distribution of extraversion and agreeableness. **(A)** Extraversion is normally distributed across all participants (left), male participants, and female participants (right). **(B)** Agreeableness is normally distributed across all participants (left), male participants, and female participants (right). The dotted line indicates the mean value of extraversion and agreeableness.

The extraversion (Figure 1A) and agreeableness scores (Figure 1B) were normally distributed across all participants (extraversion: *W* = 0.98, *p* = 0.18, agreeableness: *W* = 0.98, *p* = 0.29), across male participants (extraversion: *W* = 0.97, *p* = 0.40, agreeableness: *W* = 0.96, *p* = 0.27), and female participants (extraversion: *W* = 0.96, *p* = 0.29, agreeableness: *W* = 0.97, *p* = 0.44). There were no sex differences in extraversion [*t*(68) = 0.67, *p* = 0.5] or agreeableness [*t*(68) = 0.39, *p* = 0.7]. Mean of extraversion was 3.47 (*SD* = 0.82) for all participants, 3.41 (*SD* = 0.76) for male participants, and 3.54 (*SD* = 0.88) for female participants. Mean of agreeableness was 3.895 (*SD* = 0.59) for all participants, 3.87 (*SD* = 0.52) for male participants, and 3.92 (*SD* = 0.66) for female participants.

In our sample, mean of depression was 0.50 (*SD* = 0.32) for all participants, 0.41 (*SD* = 0.24) for male participants, and 0.59 (*SD* = 0.37) for female participants. Mean of anxiety was 0.58 (*SD* = 0.29) for all participants, 0.49 (*SD* = 0.22) for male participants, and 0.67 (*SD* = 0.33) for female participants. Female participants had greater levels of depression [*t*(68) = 2.45, *p* = 0.02] and anxiety [*t*(68) = 2.7, *p* = 0.01] compared to males. Extraversion and agreeableness were negatively associated with anxiety and depression across all participants. When separately tested for each sex, there was a significant negative association between extraversion and depression in boys and girls, and a significant negative association between agreeableness and depression in boys. See Supplementary Table 1 for statistics of the correlation analyses.

### Amygdala Connectivity

**FIGURE 2:**
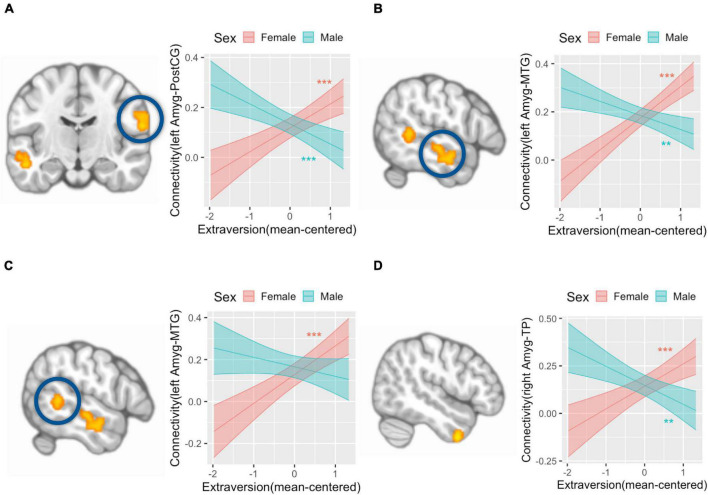
Amygdala connectivity is differentially associated with extraversion in girls and boys. Analysis with left amygdala seed region found that postcentral gyrus (**A**, left) and a region of middle temporal gyrus (MTG) (**B**, left) is associated with an interaction term of extraversion and sex in that girls showed a positive association between extraversion and connectivity, while boys showed a negative association between extraversion and connectivity (**A**, right; **B**, right). Left amygdala connectivity analysis also revealed that another region of MTG (**C**, left) is associated with the interaction term of extraversion and sex in that girls showed a positive association between extraversion and connectivity, while boys did not show a significant association between extraversion and connectivity (**C**, right). Right amygdala connectivity analysis revealed that temporal pole (**D**, left) is associated with the interaction term of extraversion by sex in that girls showed a positive association between extraversion and connectivity, while boys showed a negative association between extraversion and connectivity (**D**, right). *Y*-axis indicates predicted connectivity when the values of extraversion are entered into the multiple regression model. Asterisks indicate the significance obtained from simple slope analysis. A shaded area indicates a 95% confidence interval. **p < 0.01, ***p < 0.001; Amyg, amygdala; PostCG, postcentral gyrus; MTG, middle temporal gyrus; TP, temporal pole.

We did not find any amygdala connectivity pattern that was associated with the main effect of extraversion. The interaction between extraversion and sex was associated with left amygdala connectivity with postcentral gyrus (peak coordinate = [64, −14, 20], *k* (cluster size) = 185, *p*(FDR) < 0.001) (Figure 2A), left amygdala connectivity with two regions of middle temporal gyrus (MTG) ([−58, −18, −10], *k* = 218, *p*(FDR) < 0.001; [−58, −46, 4], *k* = 135, *p*(FDR) = 0.003) (Figures 2B,C), and right amygdala connectivity with temporal pole (TP) ([50, 6, −44], *k* = 128, *p*(FDR) = 0.01) (Figure 2D). The *post hoc* analysis to define the pattern of interaction revealed that amygdala connectivity with all four regions showed a positive association in girls (postcentral gyrus: *b* = 0.1, SE = 0.02, *p* < 0.001; TP: *b* = 0.12, SE = 0.03, *p* < 0.001; MTG([−58, −18, −10]): *b* = 0.13, SE = 0.02, *p* < 0.001; MTG([−58, −46, 4]): *b* = 0.17, SE = 0.03, *p* < 0.001). Meanwhile, amygdala connectivity with the postcentral gyrus, MTG, and TP showed a negative association with extraversion in boys (postcentral gyrus: *b* = −0.08, SE = 0.02, *p* < 0.001; MTG([−58, −18, −10]): *b* = −0.06, SE = 0.02, *p* < 0.01; TP: *b* = −0.1, SE = 0.03, *p* < 0.01). There was no association between amygdala connectivity with one of the regions in MTG ([−58, −46, 4]) and extraversion in boys (*b* = −0.04, SE = 0.03, *p* = 0.14). There was another region whose connectivity with amygdala was associated with the interaction between sex and extraversion ([2, −30, 20], *k* = 98, *p*(FDR) = 0.01), however, this region was identified as white matter. The interaction pattern of this connectivity revealed positive connectivity in boys (*b* = 0.08, SE = 0.03, *p* < 0.01) and negative connectivity in girls (*b* = −0.1, SE = 0.03, *p* < 0.001).

**FIGURE 3:**
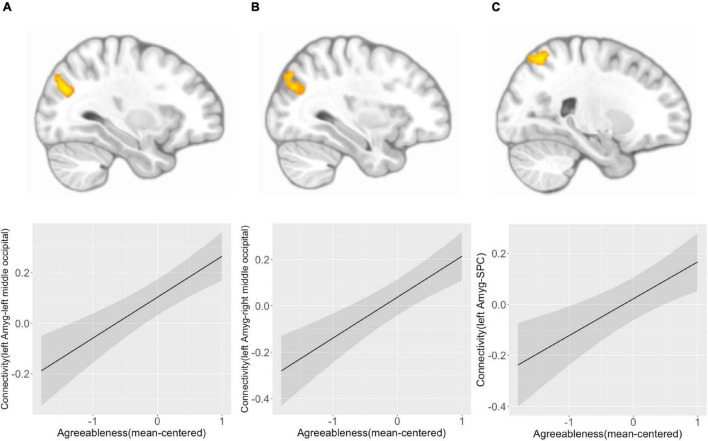
Left amygdala connectivity is positively associated with agreeableness. Agreeableness was associated with left amygdala connectivity with the left middle occipital cortex **(A)**, right middle occipital cortex **(B)**, and right superior parietal cortex **(C)**. The bottom panel plots show the pattern of association between agreeableness and amygdala connectivity with three regions. *Y*-axis indicates predicted connectivity when the values of agreeableness are entered into the multiple regression model. A shaded area indicates a 95% confidence interval. Amyg, Amygdala; SPC, superior parietal cortex.

We found that agreeableness was positively associated with left amygdala connectivity with left middle occipital cortex ([−32, −74, 28], *k* = 127, *p*(FDR) = 0.003) (Figure 3A), right middle occipital cortex ([36, −74, 28], *k* = 101, *p*(FDR) = 0.006) (Figure 3B), and right superior parietal cortex ([24, −64, 58], *k* = 185, *p*(FDR) < 0.001) (Figure 3C). Right amygdala connectivity analysis did not reveal any connectivity pattern associated with agreeableness. Both left and right amygdala connectivity analysis did not show any connectivity pattern associated with the interaction of agreeableness by sex.

### Whole-Brain Connectivity

**FIGURE 4:**
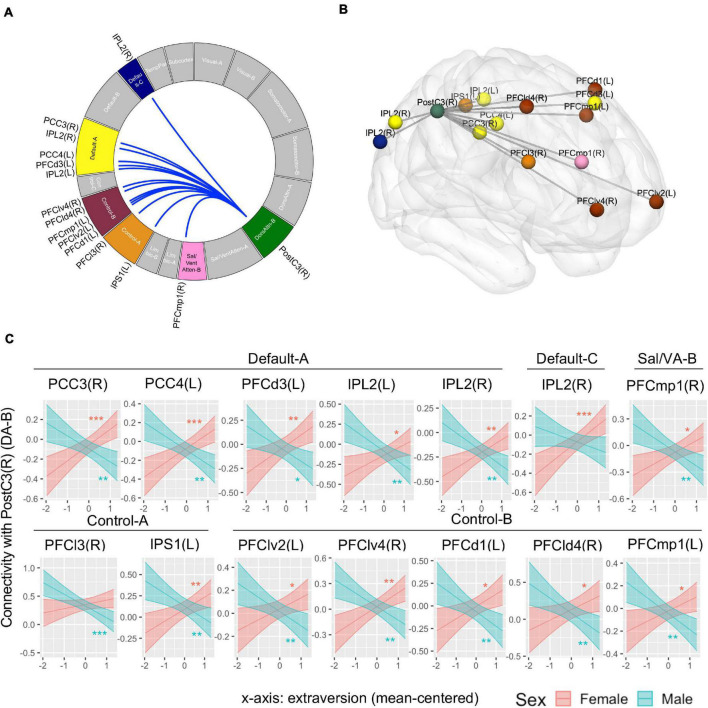
Connectivity pattern associated with the interaction term of extraversion and sex explored by whole-brain ROI-to-ROI connectivity analysis using 415 regions. **(A)** Connectome ring that represents a significant connection with a blue line. The significant networks are colored based on the previous study that introduced the brain networks and brain parcels that we used (Schaefer et al., 2018). The label of specific regions within the networks is noted outside the ring. The connectome ring was created using circa (http://omgenomics.com/circa/). **(B)** Visualization of the location of each region that showed a significant connection using BrainNet Viewer (Xia et al., 2013). The color of each region indicates the networks that each region belongs to. **(C)** The simple slope plots which demonstrate the interaction pattern of each connection. *Y*-axis indicates predicted connectivity when the values of extraversion are entered into the multiple regression model. Asterisks indicate the significance obtained from simple slope analysis. A shaded area indicates a 95% confidence interval. L, left; R, right; PCC, posterior cingulate cortex; PFCd, dorsal prefrontal cortex; IPL, inferior parietal lobule; PFCmp, posterior medial prefrontal cortex; PFCl, lateral prefrontal cortex; IPS, inferior parietal sulcus; PFClv, ventrolateral prefrontal cortex; PFCld, dorsolateral prefrontal cortex; PostC, postcentral gyrus; DA, Dorsal Attention Network; Sal/VA, Salience/Ventral Attention Network. *p < 0.05, **p < 0.01, ***p < 0.001.

Whole-brain connectivity analysis found no connection associated with the main effect of extraversion. The interaction of extraversion by sex was associated with connections of a region in DA-B network (i.e., right postcentral gyrus) [seed-level NBS statistics by intensity: 55.81, *p*(FDR) = 0.03] with regions in DMN-A, DMN-C, SAL/VA-B network, CON-A, and CON-B. The specific regions in DMN-A were right posterior cingulate cortex (PCC), left PCC, left dorsal prefrontal cortex (PFCd), left inferior parietal lobule (IPL), and right IPL. The specific region in DMN-C was the right IPL. The specific region in SAL/VA-B was the right posterior medial prefrontal cortex (PFCmp). The specific regions in CON-A were the right lateral prefrontal cortex (PFCl) and left inferior parietal sulcus (IPS). The specific regions in CON-B were left ventrolateral prefrontal cortex (PFClv), right PFClv, left PFCd, right PFCd, and left PFCmp (Figures 4A,B; see Table 1 for connection-level statistics). Simple slope analyses (Figure 4C) revealed that 12 out of 14 connections showed the same patterns of interaction in that the association between extraversion and connectivity was positive in girls, while the same association was negative in boys (see Table 1 for statistics). Two out of 14 connections showed different patterns of interaction. Specifically, postcentral gyrus connectivity with right IPL of DMN-C showed association with extraversion only in girls in the positive direction (see Table 1), and postcentral gyrus connectivity with right PFCl in CON-A showed association with extraversion only in boys in the negative direction (see Table 1).

**TABLE 1:** Functional connectivity associated with extraversion and sex interaction obtained from ROI-to-ROI analysis across 415 whole brain regions.

ROI1	ROI2	Corresponding network	Connection-level statistics	Simple slope (girls)	Simple slope (boys)
PostC (DA-B)	PCC3 (R)	DMN-A	*t*(62) = −4.58, *p*(FDR) = 0.004	*b* = 0.16***, SE = 0.04	*b* = −0.13**, SE = 0.05
	PCC4 (L)		*t*(62) = −4.70, *p*(FDR) = 0.004	*b* = 0.15***, SE = 0.04	*b* = −0.15**, SE = 0.05
	PFCd3 (L)		*t*(62) = −3.76, *p*(FDR) = 0.016	*b* = 0.15**, SE = 0.05	*b* = −0.14*, SE = 0.05
	IPL2 (L)		*t*(62) = −4.38, *p*(FDR) = 0.005	*b* = 0.13*, SE = 0.05	*b* = −0.18**, SE = 0.06
	IPL2 (R)		*t*(62) = −3.93, *p*(FDR) = 0.013	*b* = 0.13**, SE = 0.04	*b* = −0.15**, SE = 0.04
	IPL2 (R)	DMN-C	*t*(62) = −3.69, *p*(FDR) = 0.017	*b* = 0.18***, SE = 0.05	*b* = −0.08, SE = 0.05
	PFCmp1 (R)	SAL/VA-B	*t*(62) = −4.00, *p*(FDR) = 0.013	*b* = 0.13*, SE = 0.05	*b* = −0.16**, SE = 0.05
	PFCl3 (R)	CON-A	*t*(62) = −3.69, *p*(FDR) = 0.017	*b* = 0.08, SE = 0.05	*b* = −0.21***, SE = 0.06
	IPS1 (L)		*t*(62) = −3.90, *p*(FDR) = 0.013	*b* = 0.14**, SE = 0.05	*b* = −0.15**, SE = 0.05
	PFClv2 (L)	CON-B	*t*(62) = −3.52, *p*(FDR) = 0.026	*b* = 0.1*, SE = 0.04	*b* = −0.12**, SE = 0.04
	PFClv4 (R)		*t*(62) = −4.52, *p*(FDR) = 0.004	*b* = 0.16**, SE = 0.05	*b* = −0.16**, SE = 0.05
	PFCd1 (L)		*t*(62) = −3.77, *p*(FDR) = 0.016	*b* = 0.14*, SE = 0.05	*b* = −0.16**, SE = 0.06
	PFCld4 (R)		*t*(62) = −3.49, *p*(FDR) = 0.026	*b* = 0.14*, SE = 0.07	*b* = −0.2**, SE = 0.07
	PFCmp1 (L)		*t*(62) = −3.89, *p*(FDR) = 0.013	*b* = 0.09*, SE = 0.04	*b* = −0.14**, SE = 0.04

L, left; R, right; PCC, posterior cingulate cortex; PFCd, dorsal prefrontal cortex; IPL, inferior parietal lobule; PFCmp, posterior medial prefrontal cortex; PFCl, lateral prefrontal cortex; IPS, inferior parietal sulcus; PFClv, ventrolateral prefrontal cortex; PFCld, dorsolateral prefrontal cortex; PostC, postcentral gyrus; DA, Dorsal Attention Network; DMN, Default Mode Network; SAL/VA, Salience/Ventral Attention Network; CON, Control Network; b, slope coefficients; SE, Standard Error. *p < 0.05, **p < 0.01, ***p < 0.001.

There were no significant associations for the main effect of agreeableness or the interaction of agreeableness by sex that survived the correction for multiple comparisons for whole-brain connectivity.

### Within-Network Connectivity

**FIGURE 5:**
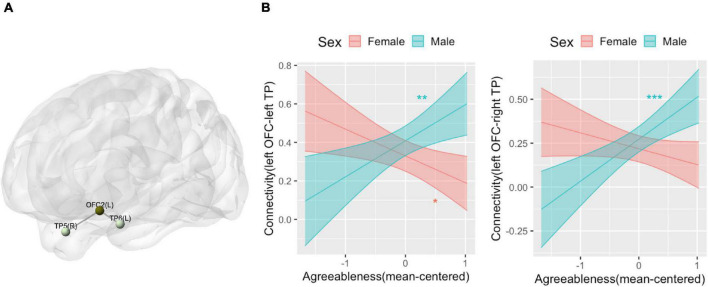
Connectivity pattern associated with the interaction term of agreeableness and sex explored by Limbic network ROI-to-ROI connectivity analysis. **(A)** Location of the orbitofrontal cortex (OFC) and bilateral temporal pole (TP) whose connectivity was associated with the interaction term of agreeableness and sex. **(B)** The interaction plots that show the interaction pattern of connectivity between OFC and left TP (left) and connectivity between OFC and right TP (right). *Y*-axis indicates predicted connectivity when the values of agreeableness are entered into the multiple regression model. Asterisks indicate the significance obtained from the simple slope analysis. A shaded area indicates a 95% confidence interval. L, left; R, right; OFC, orbitofrontal cortex; TP, temporal pole. *p < 0.05, **p < 0.01, ***p < 0.001.

We did not find any patterns of within-network connectivity surviving correction for multiple comparisons that was associated with extraversion, the extraversion by sex interaction, or agreeableness. We found that the agreeableness by sex interaction was associated with connections within the limbic network involving left OFC seed region (seed-level NBS statistics by intensity: 7.8, *p*(FDR) = 0.004), specifically, connection of left orbitofrontal cortex (OFC) with left TP [*t*(62) = 4.04, *p*(FDR) = 0.004] and right TP [*t*(62) = 3.76, *p*(FDR) = 0.004] (Figure 5A). The simple slope analysis revealed that left OFC-left TP connectivity is positively associated with agreeableness in boys (*b* = 0.19, SE = 0.07, *p* = 0.007) and it is negatively associated with agreeableness in girls (*b* = −0.14, SE = 0.06, *p* = 0.02). The left OFC-right TP connectivity is positively associated with agreeableness in boys (*b* = 0.24, SE = 0.06, *p* < 0.001) and there was no significant association in girls (*b* = −0.09, SE = 0.05, *p* = 0.1) (Figure 5B).

### Supplementary Results: Controlling for Menstrual Cycle, Depression, and Anxiety

Among 34 girls included in the final data analysis, 3 girls reported that they had not started menstruating and 3 girls skipped the questions asking about menstruation. Therefore, data from 28 girls were included for the supplementary analysis. Mean days since the last period was 16.46 (SD: 12.67, range: 0–38). When menstrual cycle was controlled for with this covariate, the female-specific results in amygdala connectivity were all replicated (Supplementary Table 2 for statistics). Female-specific findings in postcentral gyrus connectivity were partially replicated (Supplementary Table 3 for statistics). Specifically, 8 out of 13 associations remained significant. One of the two results of limbic network connectivity was replicated (Supplementary Table 4 for statistics). Specifically, the non-significant effect remained non-significant, and the one negative association became non-significant.

When we examined whether the main findings remained the same controlling for mental health outcomes (i.e., anxiety or depression) observed to be correlated with extraversion or agreeableness in our sample, we found that all results remained the same, except that the positive association between postcentral gyrus (DA-B network) to regions in Control networks [i.e., IPS1(L), PFClv2(L), PFCd1(L), PFCld4(R), PFCmp1(L)] in females became non-significant. See Supplementary Tables 5–8 for statistics.

## Discussion

The current study examined resting-state functional connectivity associated with extraversion and agreeableness in adolescence. The amygdala connectivity analysis found connectivity patterns associated with extraversion in a different direction for boys and girls. Specifically, amygdala connectivity with postcentral gyrus, MTG, and TP was positively associated with extraversion in girls, while it was negatively associated with extraversion in boys. Agreeableness was positively associated with amygdala connectivity with bilateral middle occipital cortex and right superior parietal cortex across all participants. The whole-brain connectivity analysis revealed that connectivity of a region in DA (i.e., postcentral gyrus) with regions in DMN, CON, and SAL/VA was associated with extraversion in a different direction for boys and girls, with the majority of connections showing a positive association in girls and negative association in boys. The within-network connectivity analysis showed that connections within the limbic network were positively associated with agreeableness in boys, while they showed negative associations or no association in girls. To the best of our knowledge, for the first time, our study reveals intrinsic functional connectivity associated with extraversion and agreeableness in adolescents, with a primary finding being the different direction of associations in boys and girls.

While we did not find any significant main effect of extraversion on rsFC, we observed rsFC patterns associated with the interaction of sex and extraversion. First, we found that amygdala-postcentral gyrus connectivity was positively associated with extraversion in girls, while it was negatively associated with extraversion in boys. Given that the highest proportion of this region of the postcentral gyrus belongs to the somatomotor cortex, the area responsible for integrating bodily sensation (Lloyd et al., 2015), the amygdala-postcentral gyrus connectivity could be interpreted as a neural function for generating and regulating emotion based on one’s visceral states. Indeed, it was found that amygdala-postcentral gyrus connectivity is related to emotion regulation ability (Pagliaccio et al., 2015) and this connectivity is reduced in children with a high risk for depression (Luking et al., 2011). The opposite direction of the association between amygdala-postcentral gyrus connectivity and extraversion in girls and boys could be interpreted as follows: While greater connectivity is linked to higher extraversion in girls, the same pattern of connectivity is linked to higher introversion in boys. Given that girls are more socialized to express positive emotions than boys (Chaplin, 2015), higher emotion generation and regulation associated with greater amygdala-postcentral gyrus connectivity may be related to extraverted girls’ tendency to experience and maintain positive affect, whereas the same connectivity could be related to introverted boys’ tendency to monitor their emotional responses and carefully select one’s behavior during social situations. Relatedly, it is possible that these patterns of connectivity are tapping into different facets of extraversion for boys and girls. For example, the positive association between connectivity and extraversion in girls could be driven by higher levels of the positive emotionality facet of extraversion whereas the negative association between connectivity and extraversion in boys could be driven by lower levels of gregariousness or assertiveness. Because of the relatively brief personality inventory used, we were unable to examine these facets of extraversion separately, but this would be an important direction for future research in order to test these possibilities.

Second, amygdala connectivity with two regions in MTG and TP was associated with the interaction between extraversion and sex. Amygdala connectivity with one of the MTG regions was positively associated with extraversion only in girls, whereas, amygdala connectivity within another region of MTG and TP was positively associated with extraversion in girls and negatively associated with extraversion in boys. This result is in part consistent with the findings that connectivity between the amygdala and TP is positively associated with extraversion in an adult sample (Aghajani et al., 2014; Pang et al., 2016). Additionally, the role of MTG function in individual differences in extraversion is corroborated by studies that observed the link between extraversion and MTG function measured by betweenness centrality (Gao et al., 2013), regional homogeneity (Wei et al., 2011), and activity when viewing risk-taking actions (Tamura et al., 2012).

The MTG and TP have a strong functional and anatomical connection with the amygdala and were suggested to construct a “social perception network” (Bickart et al., 2014). These regions have a crucial role in integrating social signals such as humor comprehension (Watson et al., 2007; Chan et al., 2013; Berger et al., 2018), sarcasm comprehension (Kipps et al., 2009), facial emotion recognition (Adolphs, 2002; Hennenlotter and Schroeder, 2006), face discrimination (Kogler et al., 2016), and the ability to follow a single talker against a noisy background (i.e., cocktail party effect) (Vander Ghinst et al., 2016). Interestingly, connectivity within the social perception network was found to be associated with larger social network size (Bickart et al., 2012). The observed opposite pattern based on sex is in line with the findings that the volume of medial frontal gyrus, a core region for social cognition, is associated with extraversion in boys and girls in the opposite direction (Blankstein et al., 2009). As we have speculated above, gender differences in the associations between connectivity and extraversion could be due to a variety of factors, including gender differences in the socialization of emotion expression (Chaplin, 2015) or gender differences in social cognitive abilities (Białecka-Pikul et al., 2017; Stȩpień-Nycz et al., 2020). Moreover, similar to our speculation above, these patterns of connectivity may be driven by different facets of extraversion for boys and girls.

The whole-brain network analysis found a significant interaction effect of extraversion and sex on the connectivity between a region in the DA network, specifically the postcentral gyrus, and regions in the DMN, CON, and SAL/VA network. The relevance of the attention network in extraversion is consistent with a previous study with an adult sample (Speed et al., 2015) which found that extraverts showed higher LPP or sustained attention to positive and negative stimuli. The DA is responsible for goal-directed and externally oriented perceptual attention (Corbetta and Shulman, 2002; Vossel et al., 2014). The observed relationship between extraversion and DA connectivity with three other networks may indicate that extraversion is related to the collaboration of multiple brain networks that supports directed attention to the external environment.

The DMN, SAL/VA, and CON have been suggested to have an antagonistic, complementary, and hierarchical relationship with DA for guiding complex cognition. As a task-positive and task-negative network, DA and DMN show anticorrelation during rest and tasks (Greicius et al., 2003; Fox et al., 2005; Smith et al., 2009) and their anticorrelation was found to be associated with cognitive flexibility (Owens et al., 2020). DA and DMN connectivity associated with extraversion in the current study may reflect individual differences in the tendency to suppress task-irrelevant self-generated thoughts and maintain directed attention to the external environment, such as social events. The SAL/VA is responsible for the involuntary attentional shift based on automatic bottom-up salience detection (Corbetta and Shulman, 2002) and has a complementary role with DA for attentional control (Vossel et al., 2014). The intrinsic connectivity between the regions in DA and SAL/VA may reflect the capacity to jointly utilize the two modes of attention or smoothly shift between the two modes of attention when navigating the external world. As a network with a critical role in cognitive control (Dixon and Christoff, 2012; Stokes et al., 2013) and emotion regulation (Kanske et al., 2011; Steinbeis et al., 2015), CON was suggested to regulate DA to orient attention externally in a goal-directed manner (Grady et al., 2016) and control the balance between internally oriented and externally oriented cognition (Spreng et al., 2013). Therefore, the observed CON-DA connectivity in the current study indicates that cognitive control for maintaining top-down perceptual attention and focusing on task-relevant information may be relevant to individual differences in adolescent extraversion. This finding is in line with a study that found an association between CON-DA connectivity and social inhibition (Blackford et al., 2014).

Importantly, in most connections between DA and regions in DMN, CON, and VA/SAL, we found a positive association with extraversion in girls and a negative association with extraversion in boys. Given that positive connectivity and negative connectivity are sometimes interpreted as an excitatory and inhibitory influence (Schurz et al., 2020), one possible interpretation is that DA connectivity with DMN and VA/SAL in extraverted girls and boys may reflect excitatory and inhibitory influence, respectively. For example, girls’ extraversion may be related to the greater excitatory influence of DA on DMN, while boys’ extraversion is related to the inhibitory influence of DA on DMN. However, this interpretation should be taken with caution as our rsFC analysis method did not examine the direction of connectivity. Future studies with effective connectivity analysis could test this speculation. Alternatively, as our sample of early-mid adolescents is undergoing dynamic brain and pubertal development and DA-DMN anticorrelation was found to mature during childhood and adolescence (Anderson et al., 2011), sex differences in neuromaturation in this developmental stage may underlie the sex-opposite pattern of relationships between neural connectivity across brain networks and extraversion. It should be noted that, when controlling for depression symptoms, the positive association between extraversion and DA-CON connectivity in girls became non-significant, although it remained significant for a single region in CON (i.e., right VLPFC). This may indicate that DA-CON connectivity in girls is related to the shared variance of extraversion and depression rather than a unique association with extraversion.

Across participants, agreeableness was associated with greater connectivity of the amygdala with the middle occipital cortex and superior parietal cortex. The observed relevance of the occipital cortex in agreeableness is consistent with a previous study that found an association between adolescent benevolence and surface area of the occipital cortex (Ferschmann et al., 2018) and an association between cognitive empathy and cortical thickness of the occipital cortex (Uribe et al., 2019). Furthermore, our findings are consistent with previous reports that greater basolateral amygdala connectivity with superior parietal cortex was linked to reduced aggression in adult females (Buades-Rotger et al., 2019). The amygdala and occipital cortex have a strong anatomical and functional connection, and the connectivity or coactivation of these two regions were found to support conscious and non-conscious negative memory (Steinmetz et al., 2010; Kark and Kensinger, 2015) and novelty detection (Ousdal et al., 2014), functions that help successful navigation of social worlds. Moreover, a study found that the coactivation of the amygdala and superior parietal cortex, a region responsible for visuospatial attention (Wang et al., 2015), supports the integrative process of others’ tactile sensation and facial expressions (Ebisch et al., 2016), indicating the roles of these two regions in complex social perception. Taken together, agreeableness in adolescence might be related to the neural capacity to detect, perceive, and integrate social and emotional stimuli, and these associations appear to be similar across girls and boys.

There was a sex-specific pattern of association between agreeableness and connectivity within the limbic network, specifically between OFC and the temporal pole. Only boys showed a positive correlation, and girls showed a negative or non-significant correlation. This result could be related to the finding that people with psychopathy (Craig et al., 2009) have reduced fractional anisotropy in the uncinate fasciculus, the white matter tract linking the OFC and the temporal lobe. The limbic network is known for its role in the integration of sensory stimuli to social memory (Olson et al., 2007), the integration of interoceptive and affective information to behavior (Mesulam, 2000), emotion regulation (Kanske et al., 2011), and socially appropriate behavior (Beer et al., 2006). Taken together, our results suggest that agreeableness in adolescents may be relevant to the neural capacity for learning and utilizing socioemotional information for guiding socially desirable behavior, particularly in boys. Note that we did not find patterns of connectivity that have consistently been observed to correlate with agreeableness in adult samples (i.e., DMN connectivity), suggesting that different neural networks may underlie agreeableness for adult and adolescent samples.

The results of this study should be interpreted with respect to several limitations. Specifically, one limitation is the use of a brief personality inventory, which prevented us from examining separate facets of extraversion and agreeableness. Second, the rsFC analytical approach used precluded our ability to determine the direction of connectivity, including whether patterns of observed connectivity were excitatory or inhibitory. Given these limitations, there are multiple potential explanations for the observed interactions between sex, personality, and the brain, including: (1) opposite direction of neural modulation (i.e., inhibitory influence vs. excitatory influence) is related to extraversion in boys and girls, (2) greater neural connectivity is associated with higher extraversion in girls and higher introversion in boys, (3) different facets of extraversion might have contributed to associations with functional connectivity in girls and boys. We encourage future studies to employ effective connectivity analysis, which provides the direction of neural signaling, and to examine neural connectivity associated with each facet of extraversion (e.g., sociability, assertiveness, positive emotionality) separately. Nonetheless, the results from the current study demonstrate the importance of examining sex-divergent or sex-specific patterns of neural correlates of personality, which is important particularly because most previous studies that link personality and rsFC included sex as a covariate of no interest. Third, because this study was cross-sectional, we were unable to determine the direction of effects (i.e., whether rsFC predicts differences in personality or whether differences in personality predict changes in rsFC). Fourth, it should be noted that extraversion and agreeableness are significantly correlated in our sample (*r* = 0.27), which may have led to the removal of overlapping variance because they were included as predictors in a single model. Thus, this model may not have captured connectivity associated with the shared variance between extraversion and agreeableness. Fifth, it is possible that variability in self-reported agreeableness partially reflects individual differences in socially desirable responding, as some studies have shown that agreeableness is correlated with dispositional social desirability (Graziano and Tobin, 2002; Soubelet and Salthouse, 2011; Vigil-Colet et al., 2013). Future studies using peer-reported measures of personality (e.g., Swartz et al., 2017a) or economic games that measure facets of agreeableness (e.g., trust, altruism, cooperativeness) at the behavioral level would complement our study in elucidating the neural basis of adolescent agreeableness. Finally, we could not include two adolescents with non-binary gender identities in our analysis due to the small sample size for this group, but examining these effects in adolescents with non-binary gender identities is an important direction for future research.

It should also be noted that, while we conducted a supplementary analysis that controls for girls’ menstrual cycle as a covariate, the results should be taken with caution because 6 girls out of 34 girls were not included in this analysis. Moreover, 3 girls reported that over 30 days had passed since their last period, potentially reflecting that they do not yet have a regular menstrual cycle. Nonetheless, the majority of female-specific findings remained significant, which increases our confidence that our findings are not driven by differences in girls’ menstrual cycles.

In conclusion, using an amygdala seed and whole-brain network atlas, the current study identified profiles of intrinsic functional connectivity associated with extraversion and agreeableness in adolescence, addressing the lack of studies that examine the neural basis of personality in an adolescent sample with intrinsic functional connectivity. As these two personality dimensions have a critical role in obtaining popularity and peer acceptance, the current study could help in understanding the biological basis of individual differences in forming and maintaining favorable peer relationships during adolescence. Moreover, further research into the sex-specific associations between extraversion and agreeableness in typically developing populations could help to shed light on the development of disorders associated with atypical social behavior, including autism spectrum disorders, which show marked sex differences in prevalence (Baird et al., 2006).

## Data Availability Statement

The datasets presented in this article are not readily available because requests must be sent to the corresponding author for review first. Requests to access the datasets should be directed to corresponding author.

## Ethics Statement

The studies involving human participants were reviewed and approved by the University of California, Davis Institutional Review Board. Written informed consent to participate in this study was provided by the participants’ legal guardian/next of kin.

## Author Contributions

LY and JS designed the research. AC collected the data. LY analyzed data and drafted the manuscript. JS and AC revised the manuscript. All authors contributed to the article and approved the submitted version.

## Conflict of Interest

The authors declare that the research was conducted in the absence of any commercial or financial relationships that could be construed as a potential conflict of interest.

## Publisher’s Note

All claims expressed in this article are solely those of the authors and do not necessarily represent those of their affiliated organizations, or those of the publisher, the editors and the reviewers. Any product that may be evaluated in this article, or claim that may be made by its manufacturer, is not guaranteed or endorsed by the publisher.

## Footnotes

1
https://web.conn-toolbox.org/
