# Supplementary material for: Resting-State Functional Connectivity Associated With Extraversion and Agreeableness in Adolescence
Source: Front Behav Neurosci. 2022 Jan 3;15:644790. doi: 10.3389/fnbeh.2021.644790 (PMC8762207; doi:10.3389/fnbeh.2021.644790)
Supplement: Supplementary file 1 [file Data_Sheet_1.docx]

Supplementary Material

**Supplementary Table 1. Correlation between personality measures (i.e., extraversion, agreeableness) and mental health symptoms (i.e., anxiety, depression).**

|  | Whole Sample (N=70) | Female (N=34) | Male (N=36) |
| --- | --- | --- | --- |
| Extraversion - Anxiety | **r = -.24, *p* = .04** | r = -.29, *p* = .1 | r = -.28, *p* = .1 |
| Extraversion - Depression | **r = -.32, *p* = .01** | **r = -.37, *p* = .03** | **r = -.35, *p* = .04** |
| Agreeableness - Anxiety | **r = -.27, *p* = .02** | r = -.29, *p* = .1 | r = -.33, *p* = .051 |
| Agreeableness - Depression | **r = -.34, *p* = .004** | r = -.34, *p* = .052 | **r = -.44, *p* = .01** |

**Supplementary Table 2. The association between extraversion and amygdala connectivity in girls controlling for menstrual cycle measured by days since last period.** amyg, amygdala; postCG, postcentral gyrus; MTG, middle temporal gyrus; TP, temporal pole; b, slope coefficients; SE, Standard Error. * *p*<.05, *** *p*<.001

| connectivity | Original Result | Result after controlling for menstrual cycle | Statistics |
| --- | --- | --- | --- |
| left amyg - postCG | positive | positive | b = 0.1***, SE = 0.02 |
| left amyg - MTG ([-58, -18, -10]) | positive | positive | b = 0.14***, SE = 0.02 |
| left amyg - MTG ([-58, -46, 4]) | positive | positive | b = 0.12***, SE = 0.02 |
| right amyg - TP | positive | positive | b = 0.1*, SE = 0.04 |

**Supplementary Table 3. The association between extraversion and postcentral gyrus connectivity in girls controlling for menstrual cycle measured by days since last period.** The results that are inconsistent with the original findings are underlined. L, left; R, right; PCC, posterior cingulate cortex; PFCd, dorsal prefrontal cortex; IPL, inferior parietal lobule; PFCmp, posterior medial prefrontal cortex; PFCl, lateral prefrontal cortex; IPS, inferior parietal sulcus; PFClv, ventrolateral prefrontal cortex; PFCld, dorsolateral prefrontal cortex; PostC, postcentral gyrus; DA, Dorsal Attention Network; DMN, Default Mode Network; SAL/VA, Salience/Ventral Attention Network; CON, Control Network; b, slope coefficients; SE, Standard Error; NS, Not Significant. * *p*<.05

| ROI1 | ROI2 | Corresponding network | Original Result | Result after controlling for menstrual cycle | Statistics |
| --- | --- | --- | --- | --- | --- |
| PostC  (DA-B) | PCC3 (R) | DMN-A | positive | positive | b = 0.13*, SE = 0.05 |
|  | PCC4 (R) |  | positive | positive | b = 0.13*, SE = 0.05 |
|  | PFCd3 (L) |  | positive | positive | b = 0.15*, SE = 0.06 |
|  | IPL2 (L) |  | positive | positive | b = 0.15*, SE = 0.06 |
|  | IPL2 (R) |  | positive | positive | b = 0.13*, SE = 0.05 |
|  | IPL2 (R) | DMN-C | positive | positive | b = 0.16*, SE = 0.07 |
|  | PFCmp1 (R) | SAL/VA-B | positive | marginally  positive (*p* = 0.066) | b = 0.14, SE = 0.07 |
|  | PFCl3 (R) | CON-A | NS | NS | b = 0.1, SE = 0.07 |
|  | IPS1 (L) |  | positive | NS | b = 0.11, SE = 0.07 |
|  | PFClv2 (L) | CON-B | positive | NS | b = 0.1, SE = 0.06 |
|  | PFClv4 (R) |  | positive | positive | b = 0.14*, SE = 0.06 |
|  | PFCd1 (L) |  | positive | positive | b = 0.15*, SE = 0.06 |
|  | PFCld4 (R) |  | positive | marginally  positive (*p* = 0.06) | b = 0.18, SE = 0.09 |
|  | PFCmp1 (L) |  | positive | NS | b = 0.09, SE = 0.06 |

**Supplementary Table 4. The association between agreeableness and within-limbic connectivity in girls controlling for menstrual cycle measured by days since last period.** The results that are inconsistent with the original findings are underlined. OFC, orbitofrontal cortex; TP, temporal pole; b, slope coefficients; SE, Standard Error; NS, Not Significant.

| connectivity | Original Result | Result after controlling for menstrual cycle | Statistics |
| --- | --- | --- | --- |
| left OFC - left TP | negative | NS | b = -0.1, SE = 0.06 |
| left OFC - right TP | NS | NS | b = -0.08, SE = 0.05 |

**Supplementary Table 5. The association between extraversion and amygdala connectivity controlling for depression.** Multiple regressions were tested separately for boys and girls. Independent variables included extraversion, agreeableness, depression, age, and scan length. amyg, amygdala; postCG, postcentral gyrus; MTG, middle temporal gyrus; TP, temporal pole; b, slope coefficients; SE, Standard Error. * *p*<.05, ** *p*<.01, *** *p*<.001

| connectivity | Original Result | Result after controlling for depression | Statistics |
| --- | --- | --- | --- |
| Female | | | |
| left amyg - postCG | positive | positive | b = 0.11***, SE = 0.02 |
| left amyg - MTG ([-58, -18, -10]) | positive | positive | b = 0.13***, SE = 0.02 |
| left amyg - MTG ([-58, -46, 4]) | positive | positive | b = 0.18***, SE = 0.03 |
| right amyg - TP | positive | positive | b = 0.12**, SE = 0.04 |
| Male | | | |
| left amyg - postCG | negative | negative | b = -0.06*, SE = 0.03 |
| left amyg - MTG ([-58, -18, -10]) | negative | negative | b = -0.06*, SE = 0.02 |
| left amyg - MTG ([-58, -46, 4]) | NS | NS | b = -0.04, SE = 0.03 |
| right amyg - TP | negative | negative | b = -0.1**, SE = 0.03 |

**Supplementary Table 6. The association between agreeableness and amygdala connectivity across full sample controlling for depression and anxiety.** Independent variables of multiple regressions included extraversion, agreeableness, sex, extraversion*sex, agreeableness*sex, depression, anxiety, age, and scan length. amyg, amygdala; postCG, postcentral gyrus; MTG, middle temporal gyrus; TP, temporal pole; b, slope coefficients; SE, Standard Error. ** *p*<.01, *** *p*<.001

| connectivity | Original Result | Result after controlling for depression and anxiety | Statistics |
| --- | --- | --- | --- |
| left amyg - SPL | positive | positive | b = 0.14**, SE = 0.04 |
| left amyg – left Occipital cortex | positive | positive | b = 0.16***, SE = 0.03 |
| left amyg – right Occipital cortex | positive | positive | b = 0.17***, SE = 0.04 |

**Supplementary Table 7. The association between extraversion and postcentral gyrus connectivity controlling for depression.** Multiple regressions were tested separately for boys and girls. Independent variables included extraversion, agreeableness, depression, age, and scan length. The results that are inconsistent with the original findings are underlined. L, left; R, right; PCC, posterior cingulate cortex; PFCd, dorsal prefrontal cortex; IPL, inferior parietal lobule; PFCmp, posterior medial prefrontal cortex; PFCl, lateral prefrontal cortex; IPS, inferior parietal sulcus; PFClv, ventrolateral prefrontal cortex; PFCld, dorsolateral prefrontal cortex; PostC, postcentral gyrus; DA, Dorsal Attention Network; DMN, Default Mode Network; SAL/VA, Salience/Ventral Attention Network; CON, Control Network; b, slope coefficients; SE, Standard Error; NS, Not Significant. * *p*<.05, ** *p*<.01, *** *p*<.001

| ROI1 | ROI2 | Corresponding network | Original Result | Result after controlling for depression | Statistics |
| --- | --- | --- | --- | --- | --- |
| Female | | | | | |
| PostC  (DA-B) | PCC3 (R) | DMN-A | positive | positive | b = 0.16**, SE = 0.05 |
|  | PCC4 (R) |  | positive | positive | b = 0.16**, SE = 0.05 |
|  | PFCd3 (L) |  | positive | positive | b = 0.16*, SE = 0.06 |
|  | IPL2 (L) |  | positive | positive | b = 0.12*, SE = 0.05 |
|  | IPL2 (R) |  | positive | positive | b = 0.1*, SE = 0.04 |
|  | IPL2 (R) | DMN-C | positive | positive | b = 0.19**, SE = 0.06 |
|  | PFCmp1 (R) | SAL/VA-B | positive | positive | b = 0.15*, SE = 0.06 |
|  | PFCl3 (R) | CON-A | NS | NS | b = 0.06, SE = 0.06 |
|  | IPS1 (L) |  | positive | NS | b = 0.1, SE = 0.06 |
|  | PFClv2 (L) | CON-B | positive | NS | b = 0.08, SE = 0.05 |
|  | PFClv4 (R) |  | positive | positive | b = 0.16**, SE = 0.06 |
|  | PFCd1 (L) |  | positive | NS | b = 0.11, SE = 0.06 |
|  | PFCld4 (R) |  | positive | NS | b = 0.12, SE = 0.08 |
|  | PFCmp1 (L) |  | positive | NS | b = 0.05, SE = 0.05 |
| Male | | | | | |
| PostC  (DA-B) | PCC3 (R) | DMN-A | negative | negative | b = -0.12**, SE = 0.05 |
|  | PCC4 (R) |  | negative | negative | b = -0.14**, SE = 0.05 |
|  | PFCd3 (L) |  | negative | negative | b = -0.13*, SE = 0.06 |
|  | IPL2 (L) |  | negative | negative | b = -0.15*, SE = 0.07 |
|  | IPL2 (R) |  | negative | negative | b = -0.15**, SE = 0.05 |
|  | IPL2 (R) | DMN-C | NS | NS | b = -0.1, SE = 0.05 |
|  | PFCmp1 (R) | SAL/VA-B | negative | negative | b = -0.15**, SE = 0.05 |
|  | PFCl3 (R) | CON-A | negative | negative | b = -0.21***, SE = 0.05 |
|  | IPS1 (L) |  | negative | negative | b = -0.16**, SE = 0.05 |
|  | PFClv2 (L) | CON-B | negative | negative | b = -0.11*, SE = 0.04 |
|  | PFClv4 (R) |  | negative | negative | b = -0.16**, SE = 0.05 |
|  | PFCd1 (L) |  | negative | negative | b = -0.18**, SE = 0.06 |
|  | PFCld4 (R) |  | negative | negative | b = -0.19**, SE = 0.07 |
|  | PFCmp1 (L) |  | negative | negative | b = -0.15***, SE = 0.04 |

**Supplementary Table 8. The association between agreeableness and within-limbic connectivity controlling for depression.** Multiple regressions were tested only for boys given that the correlation between agreeableness and depression was significant only in boys. Independent variables included agreeableness, extraversion, depression, age, and scan length. OFC, orbitofrontal cortex; TP, temporal pole; b, slope coefficients; SE, Standard Error; NS, Not Significant. * *p*<.05, ** *p*<.01

| connectivity | Original Result | Result after controlling for depression | Statistics |
| --- | --- | --- | --- |
| left OFC - left TP | positive | positive | b = 0.19*, SE = 0.08 |
| left OFC - right TP | positive | positive | b = 0.2**, SE = 0.08 |

**Supplementary Figure 1. 17 networks (Schaefer et al., 2018) and subcortex (provided by CONN toolbox) used for whole-brain connectivity analysis.** Please refer the full list of 400 ROIs of 17 networks that we used at <https://github.com/ThomasYeoLab/CBIG/blob/master/stable_projects/brain_parcellation/Schaefer2018_LocalGlobal/Parcellations/MNI/Centroid_coordinates/Schaefer2018_400Parcels_17Networks_order_FSLMNI152_2mm.Centroid_RAS.csv>.


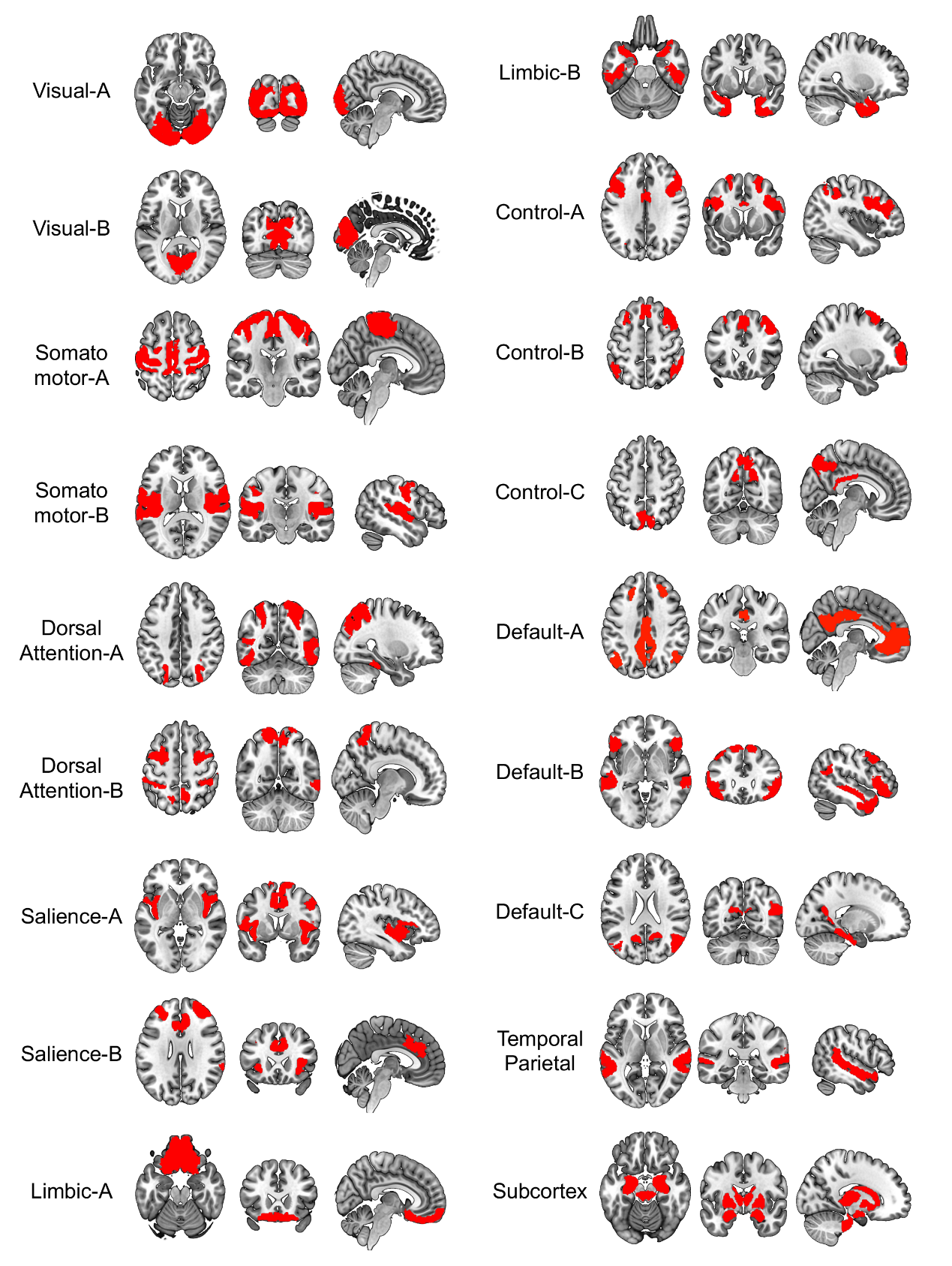


**References**

Schaefer, A., Kong, R., Gordon, E.M., Laumann, T.O., Zuo, X.-N., Holmes, A.J., et al. (2018). Local-global parcellation of the human cerebral cortex from intrinsic functional connectivity MRI. *Cerebral cortex* 28(9)**,** 3095-3114.
